# Do patient-reported outcome scores better identify outlier surgical practice compared with revision rates for total knee arthroplasty?

**DOI:** 10.2340/17453674.2025.44037

**Published:** 2025-06-25

**Authors:** Morgan C H LINGARD, Christopher M A FRAMPTON, Gary J HOOPER

**Affiliations:** 1 Department of Orthopaedic Surgery and Musculoskeletal Medicine, University of Otago, Christchurch; 2 Department of Medicine, University of Otago, Christchurch, New Zealand

## Abstract

**Background and purpose:**

The New Zealand Joint Registry provides surgeon-level feedback on revision rate and Oxford scores for primary total knee replacement (TKR). Potential outliers are identified using revision rate. Using patient-reported outcome measures to identify outliers, alongside revision rate, could provide a more comprehensive understanding of surgeons’ results. We aimed to evaluate using Oxford scores compared with revision rates to identify potential outliers.

**Methods:**

A registry-based prospective longitudinal cohort design was used. The association between surgeon mean Oxford score at 6 months and revision rate at 2 years, within 5 years, and within 10 years was evaluated using the Pearson correlation coefficient. Funnel and scatter plots were used to compare potential outliers for both measures using control limit and centile outlier thresholds respectively. All TKR in the registry prior to 31 December 2021, performed for any indication, were included.

**Results:**

There was a weak negative association between mean Oxford score at 6 months and revision rate at 2 years, within 5 years, and within 10 years using the Pearson correlation coefficient. Funnel plot control limits identified similar numbers of outliers for 6-month Oxford score and revision rate, however, there here was minimal overlap in outliers identified using the 2 methods. There was also minimal overlap in outliers using centile thresholds.

**Conclusion:**

Correlation between Oxford score at 6 months and revision rates is weaker at the surgeon level compared with the patient level. Mean Oxford score identifies different potential outliers compared with revision rates with minimal overlap. This has implications for reporting surgeon-level outcomes, raising questions regarding the most appropriate measure of surgical performance following TKR.

Surgeon-specific outcome monitoring has been applied to joint replacement registries to reduce poor outcomes for patients. The New Zealand Orthopaedic Association monitors individual arthroplasty surgeon performance using revision rates derived from the New Zealand Joint Registry (NZJR).

Annual reports are provided confidentially to surgeons including revision rates presented on funnel plots. Surgeons above the 95% control limit are identified as potential outliers and required to complete further audit and present the findings to their peers, whereby suggestions are made to improve performance. Surgeons receive feedback on Oxford scores; however, these are not formally compared with their peers and surgeons cannot be identified as outliers using Oxford scores.

Surgeon-specific outcome monitoring remains contentious. However, when provided in a confidential manner, surgeons tend to support its use [1]. Monitoring performance provides quality assurance and has the potential to improve outcomes. A recent systematic review published on the effect of feedback on surgical outcomes found that feedback tended to improve subsequent performance [2]. While revision rates have historically been used for this purpose by our organization, Oxford scores could be feasibly incorporated when assessing surgeon performance.

The 6-month Oxford score has been identified as a reliable PROM for identifying potentially failing prostheses. Scores less than 27 are associated with significant risk of revision within 2 years and this observation has remained valid out to 10 years [3].

The motivation for this study was to establish whether assessment of surgeon performance could be improved with the use of patient-reporting outcome measures. We aimed to evaluate the relationship between 6-month Oxford score and revision rate at selected timeframes, and compare outliers identified using each measure for total knee replacement (TKR).

## Methods

### Study design

This study used a registry-based prospective longitudinal cohort design. The relationship between Oxford score and revision rate was compared at the surgeon level across multiple reporting timeframes. The STROBE guideline was used for reporting [4].

### Data source

Analysis was completed using data from the New Zealand Joint Registry, which has near-complete capture of primary knee joint replacement procedures and revisions in New Zealand [5]. Revision is defined by the registry as reoperation with exchange of a component. The registry distributes 6-month Oxford knee scores to a random selection of patients, targeting a response rate of 20% [6,7]. Questionnaires are sent to 27% of patients, on average, to achieve this. NZJR also collects 5-year, 10-year, 15-year, and 20-year Oxford scores. Preoperative Oxford scores are not available.

### Patients

Reporting timeframes of revision at 2 years, within 5 years, and within 10 years were selected to represent short- to long-term outcomes and were being considered for surgeon-level reporting by our organization. The revision at 2 years’ timeframe required included procedures to have 2 years of complete follow-up and was calculated per procedure, whereas the revisions within 5 and 10 years’ timeframes included more recent procedures and were calculated per component year. A joint contributed component years until it was either revised, the patient died, or follow-up to the defined end-point was completed. Revision at 2 years included procedures from 1 January 2015 to 31 December 2019, representing 5 years of procedures with completed follow-up. Revision within 5 years included procedures from 1sJanuary 2012 to 31 December 2021 and revision within 10 years from 1 January 2002 to 31 December 2021, representing 5 and 10 years of procedures with completed follow-up respectively. Date ranges for each timeframe were those which would have been included for reports on the 31 December 2021.

All procedures performed within the date range for each timeframe were included in that analysis. For patient-level analyses, procedures without an Oxford score were excluded. For surgeon-level analyses, surgeons were included if they had performed at least 1 TKR in the 2 years preceding 31December 2021 and at least 1 Oxford score response in the reporting timeframe. Procedures performed by surgeons who did not meet these criteria were excluded. Other than this, no exclusion criteria were applied.

### Statistics

The Pearson correlation coefficient was used to analyze the association between surgeon-level mean Oxford score at 6 months and revision rate at 2 years, within 5 years, and within 10 years. The association between mean Oxford score at 6 months and revision rate at 2 years was evaluated at the patient level using logistic regression analysis for TKR performed between 1 January 2010 and 31 December 2019, to demonstrate that the previously established association was present in our dataset.

Scatter plots for mean Oxford score vs revision rate were constructed and potential outliers compared using the 97.5th and 90th centile for revision rate and the 2.5th and 10th centile for mean 6-month Oxford score respectively. Funnel plots for revision rate and Oxford score at 6 months were then constructed and 95% control limits used to identify potential outliers. Surgeons identified as having revision rates above the 95% control limit and mean Oxford scores below the 95% control limit were compared. These 2 methods for defining limits of acceptable performance were similar, but differed in that centiles are strongly empirical while the control limit approach acknowledges greater uncertainty associated with lower procedure volumes. The 97.5th and 2.5th centiles and 95% control limits represented the equivalent threshold for the 2 methodologies, while the 90th and 10th centiles were included for comparison, as the higher centile threshold identified very few surgeons as outliers. The comparison using centile outlier thresholds and funnel plot control limits was then completed for revision within 10 years vs 5-year Oxford score to evaluate whether the findings applied to later Oxford scores; the 5-year Oxford score is the second time-point collected by NZJR.

The distribution of demographic factors, preoperative diagnosis, and fixation technique was then evaluated for outliers for revision rate and Oxford score using funnel plot control limits. NZJR collects data on age, sex, ethnicity, body mass index (BMI), and American Association of Anesthesiologists (ASA) class. Total knee arthroplasty fixation was classified as cemented, uncemented, or hybrid. Statistical significance of differences between outlier surgeons and those below the outlier threshold was assessed using the z-test for independent proportions.

It was recognized that, for scatter plots, low-volume surgeons had the potential to be spurious outliers. For funnel plots, variation in outlier threshold by surgical volume accounted for this, making this our preferred method for monitoring surgeon-level performance.

Statistical analysis was completed using SPSS statistics (version 29; IBM Corp, Armonk, NY, USA).

### Ethics, funding, use of AI, and disclosures

No grants or other support were required for this study. Gary Hooper is chairman of the board of trustees for the New Zealand Joint Registry. AI tools were not used. No other conflicts of interest were declared. Complete disclosure of interest forms according to ICMJE are available on the article page, doi: 10.2340/17453674.2025.44037

## Results

**Figure 1:**
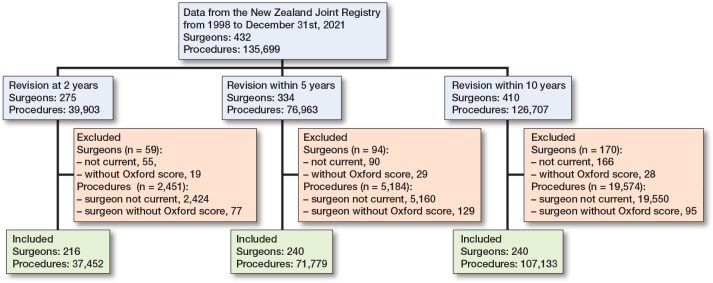
Flowchart for surgeons and procedures included in surgeon-level analysis. Current surgeons were defined as having completed at least 1 procedure from 1 January 2020 to 31 December 2021.

Figure 1 presents the flowchart for surgeons and procedures included in the surgeon-level analysis. There were 432 surgeons and 135,699 procedures recorded in the registry on 31 December 2021. 216 surgeons were included for the revision at 2 years’ timeframe, while 240 surgeons were included for the revision within the 5 and 10 years’ timeframes. A substantial proportion of surgeons in the registry had not performed a procedure in the previous 2 years, or did not have a single 6-month Oxford score. However, these surgeons accounted for a relatively small proportion of procedures for each timeframe.

### Correlation between Oxford score and revision

Table 1 summarizes the number of surgeons and the median number of procedures, component years, number of Oxford score responses, Oxford score, and revision rate for the selected reporting timeframes. At the surgeon level, there was a weak but statistically significant negative association between mean 6-month Oxford score and revision rate for all included reporting timeframes. The Pearson correlation coefficient was –0.16 (P = 0.02) for revision at 2 years, –0.24 (P < 0.001) for within 5 years, and –0.23 (P < 0.001) for within 10 years.

**Table 1:** Characteristics of reporting timeframes

	Revision at 2 years	Revision within 5 years	Revision within 10 years
Surgeons	216	240	240
Median procedures	131.5	216	316
Median component years	–	771	1,676
Median Oxford responses	21	38	59.5
Median revision rate	0.0122**^a^**	0.00539**^b^**	0.00470**^b^**
Median Oxford score	38.5	38.1	37.9

a per procedure

b per component year

At the patient level, there was a statistically significant negative association between mean 6-month Oxford score and the likelihood of revision at 2 years for procedures performed between 1 January 2010 and 31 December 2019. For every 1 point decrease in Oxford score, risk of revision increased by 11.3% (P < 0.001). At the surgeon level, there was a weak but statistically significant association between mean 6-month Oxford score and percentage revised at 2 years for this date range (Pearson correlation coefficient –0.17, P = 0.01).

### Scatter plots Oxford score vs revision rate

**Figure 2:**
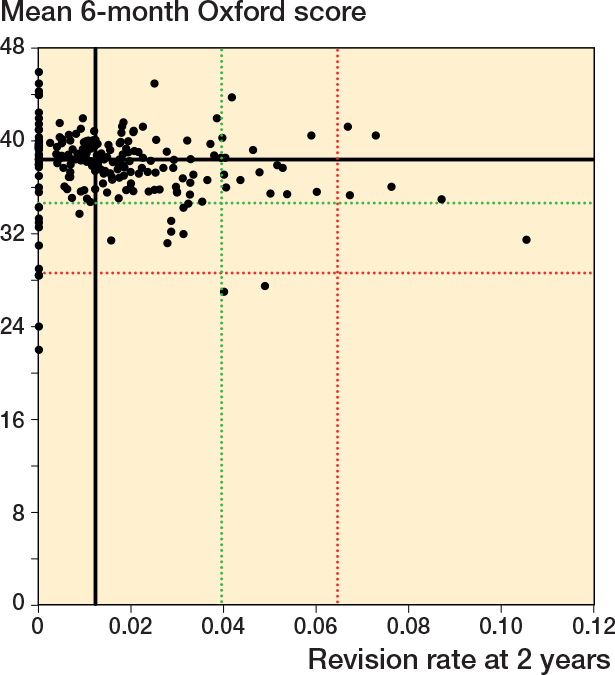
Scatter plot of revision rate at 2 years vs mean Oxford score at 6 months. Black line = median, red line = 97.5th centile for revision rate and 2.5th centile for Oxford score, green line = 90th centile for revision rate and 10th centile for Oxford score.

**Figure 3:**
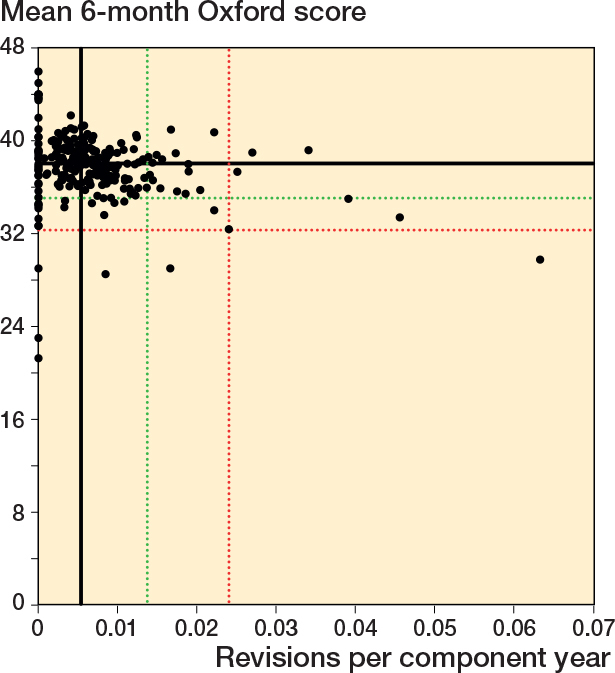
Scatter plot of revision rate within 5 years vs mean Oxford score at 6 months. See Figure 2 for explanation.

**Figure 4:**
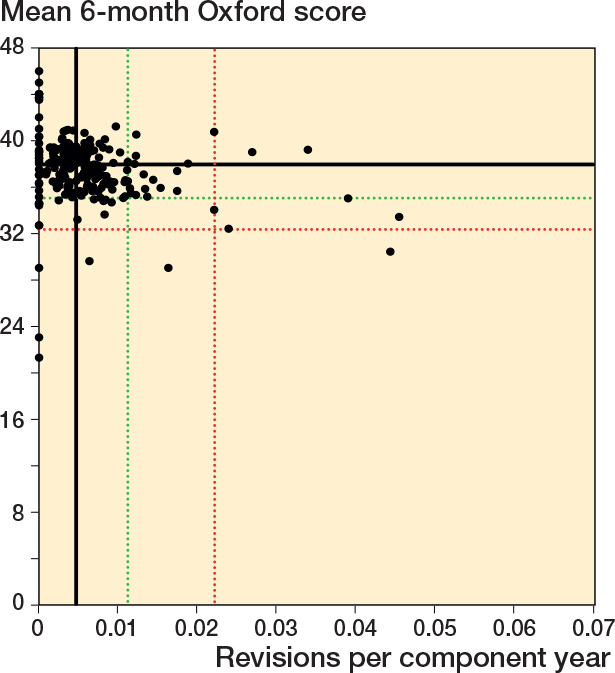
Scatter plot of revision rate within 10 years vs mean Oxford score at 6 months. See Figure 2 for explanation.

For all timeframes, there was no clear association between surgeon mean 6-month Oxford score and revision rate (Figures 2 to 4). There was minimal overlap between potential outliers using centile outlier thresholds. The distribution of surgeons on the scatter plots is summarized in Table 2.

**Table 2:** Potential outliers for total knee arthroplasty revision rate vs 6-month Oxford score using scatter plots and centile outlier thresholds. Surgeons were identified as outliers using the 90th and 97.5th centiles for revision rate and 10th and 2.5th centiles for 6-month Oxford score. Outliers at the higher threshold for each measure also are outliers at the lower threshold. Values are count

6-month Oxford score (centile)	Revision at 2 years (centile)			
	≥ 97.5th	≥ 90th	< 90th	Total
≤ 2.5th	0	2	4	
≤ 10th	1	3	19	
> 10th	5	19	175	
Total				216
6-month Oxford score (centile)	Revision within 5 years (centile)			
	≥ 97.5th	≥ 90th	< 90th	Total
≤ 2.5th	1	2	4	
≤ 10th	3	6	18	
> 10th	3	18	198	
Total				240
6-month Oxford score (centile)	Revision within 10 years (centile)			
	≥ 97.5th	≥ 90th	< 90th	Total
≤ 2.5th	1	2	4	
≤ 10th	4	6	18	
> 10th	2	18	198	
Total				240

For revision at 2 years, 41 of 216 surgeons had a revision rate greater than or equal to the 90th centile and/or a mean Oxford score at 6 months equal to or less than the 10th centile; 3 surgeons were outliers for both measures. 12 surgeons had either a revision rate greater than or equal to the 97.5th centile and/or a mean Oxford score at 6 months equal to or less than the 2.5th centile. There was no overlap in potential outliers at this threshold. The lower number of surgeons included in this analysis, compared with the other timeframes, was due to a small number of surgeons who were only active after 31 December 2019.

For revision within 5 years, 42 of 240 surgeons had a revision rate greater than or equal to the 90th centile and/or a mean Oxford score at 6 months equal to or less than the 10th centile; 6 surgeons were potential outliers for both measures. 11 surgeons had either a revision rate greater than or equal to the 97.5th centile and/or a mean Oxford score at 6 months equal to or less than the 2.5th centile. 1 surgeon was a potential outlier for both measures at this threshold.

For revision within 10 years, 42 of 240 surgeons had a revision rate greater than or equal to the 90th centile and/or a mean Oxford score at 6 months equal to or less than the 10th centile; 6 surgeons were potential outliers for both measures. 11 surgeons had either a revision rate greater than or equal to the 97.5th centile and/or a mean Oxford score at 6 months equal to or less than the 2.5th centile. 1 surgeon was a potential outlier for both measures at this threshold.

### Funnel plots Oxford score vs revision rate

**Figure 5:**
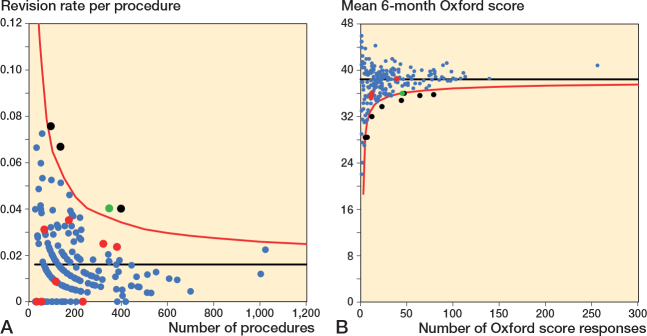
Funnel plots for revision rate at 2 years and mean 6-month Oxford score. A. Revision rate at 2 years. Funnel plot presenting the performance of individual surgeons for revision rate. B. Mean Oxford score at 6 months. Funnel plot presenting the performance of individual surgeons for Oxford score. The black line is the target and red line the outlier control limit. Individual surgeons are represented by dots. Green dot = outlier for both revision rate and Oxford score, black dot = outlier for Oxford score only, and red dot = outlier for revision rate only.

**Figure 6:**
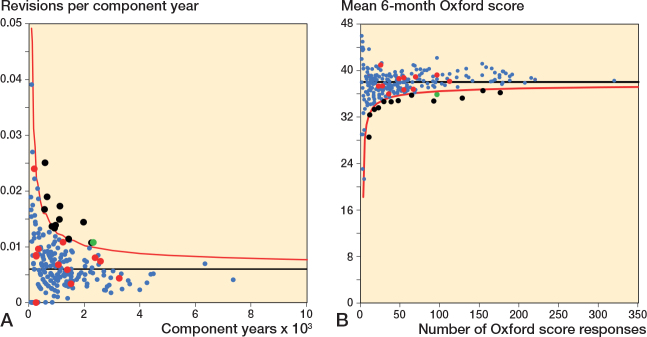
Funnel plots for revision rate within 5 years and mean 6-month Oxford score. A. Revision rate within 5 years. B. Mean Oxford score at 6 months. See Figure 5 for explanations.

**Figure 7:**
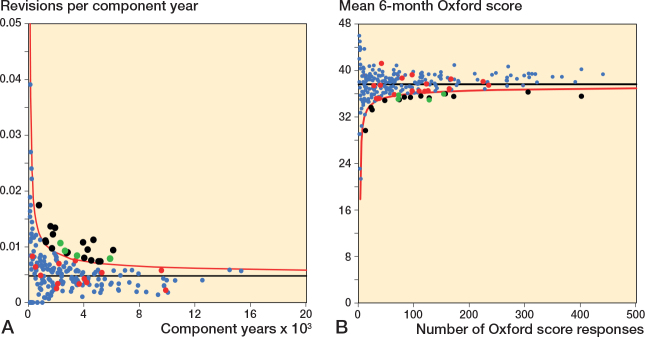
Funnel plots for revision rate within 10 years and mean 6-month Oxford score. A. Revision rate within 10 years. B. Mean Oxford score at 6 months. See Figure 5 for explanations.

Funnel plots for revision rate at 2 years, within 5 years, and within 10 years showed minimal overlap in potential outliers using revision rate and mean 6-month Oxford score (Figures 5 to 7). Table 3 summarizes the distribution of surgeons using control limit outlier thresholds for each timeframe.

**Table 3:** Potential outliers for total knee arthroplasty revision rate vs 6-month Oxford score using funnel plot control limit outlier thresholds. Surgeons were identified as outliers using the 95% upper control limit for revision rate and the 95% lower control limit for 6-month Oxford score. Values are count

6-month Oxford score	Revision at 2 years		Total
	exceeds upper control limit	within upper control limit	
Below lower control limit	1	8	9
Within control limit	3	204	207
Total	4	212	216
6-month Oxford score	Revision within 5 years		Total
	exceeds upper control limit	within upper control limit	
Below lower control limit	1	12	13
Within control limit	11	216	227
Total	12	228	240
6-month Oxford score	Revision within 10 years		Total
	exceeds upper control limit	within upper control limit	
Below lower control limit	4	13	17
Within control limit	17	206	223
Total	21	219	240

For revision at 2 years, 9 of 216 surgeons were identified as potential outliers using the 6-month Oxford score funnel plot compared with 4 for revision rate. 1 surgeon was identified as a potential outlier using both mean Oxford score at 6 months and revision rate at 2 years.

For revision within 5 years, 13 of 240 surgeons were identified as potential outliers using the 6-month Oxford score funnel plot compared with 12 for revision rate. 1 surgeon was identified as a potential outlier using both mean Oxford score at 6 months and revision rate within 5 years.

For revision within 10 years, 17 of 240 surgeons were identified as potential outliers using the 6-month Oxford score funnel plot compared with 21 for revision rate. 4 surgeons were identified as a potential outliers using both mean Oxford score at 6 months and revision rate within 10 years.

### 5-year Oxford scores

**Figure 8:**
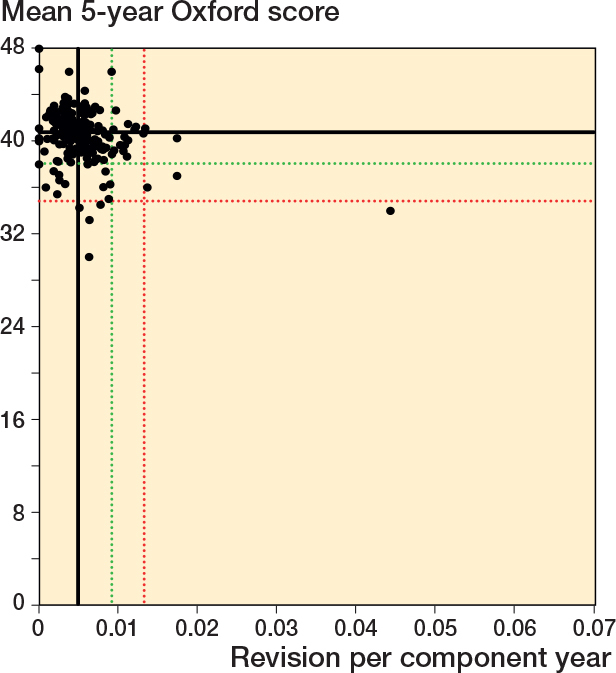
Scatter plot of revision rate within 10 years vs mean Oxford score at 5 years. See Figure 2 for explanation.

**Figure 9:**
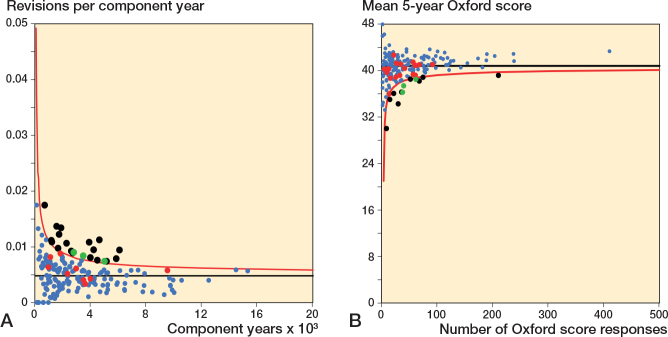
Funnel plots for revision rate within 10 years and mean 5-year Oxford score. A. Revision rate within 10 years. B. Mean Oxford score at 5 years. See Figure 5 for explanations.

At the surgeon level, there was a weak but statistically significant association between mean 5-year Oxford score and revision rate within 10 years. The Pearson correlation coefficient was –0.31 (P < 0.001). Figures 8 and 9 (see Appendix) present the scatter and funnel plots for revision rate within 10 years vs 5-year Oxford score. To be included in this analysis, surgeons needed to have at least 1 x 5-year Oxford score response, resulting in fewer being included. Tables 4 and 5 (see Appendix) summarize the distribution of surgeons using centile and control limit outlier thresholds respectively. For both methods, there was minimal overlap in potential outliers.

**Table 4:** Potential outliers for total knee arthroplasty revision rate within 10 years vs 5-year Oxford score using scatter plots and centile outlier thresholds. Surgeons were identified as outliers using the 90th and 97.5th centiles for revision rate and 10th and 2.5th centiles for 5-year Oxford score. Outliers at the higher threshold for each measure also are outliers at the lower threshold Values are count

5-year Oxford score (centile)	Revision rate within 10 years (centile)			
	≥ 97.5th	≥ 90th	< 90th	Total
≤ 2.5th	1	0	4	
≤ 10th	3	3	16	
> 10th	2	16	151	
Total				186

**Table 5:** Potential outliers for total knee arthroplasty revision rate within 10 years vs 5-year Oxford score using funnel plot control limit outlier thresholds. Surgeons were identified as outliers using the 95% upper control limit for revision rate and the 95% lower control limit for 5-year Oxford score. Values are count

6-year Oxford score	Revision rate within 10 years		
	exceeds upper control limit	within upper control limit	Total
Below lower control limit	3	9	12
Within control limit	18	156	174
Total	21	165	186

35 of 186 surgeons had a revision rate greater than or equal to the 90th centile and/or a mean Oxford score at 5 years equal to or less than the 10th centile; 3 surgeons were outliers for both measures. 9 surgeons had either a revision rate greater than or equal to the 97.5th centile and/or a mean Oxford score at 5 years equal to or less than the 2.5th centile. 1 surgeon was a potential outlier for both measures at this threshold.

12 of 186 surgeons were identified as potential outliers using the 5-year Oxford score funnel plot compared with 21 for revision rate within 10 years. Three surgeons were identified as a potential outlier using both mean Oxford score at 5 years and revision rate within 10 years.

### Distribution of demographic factors, preoperative diagnosis and fixation technique

Tables 6 to 8 (see Appendix) demonstrate the distribution of demographic factors, preoperative diagnosis, and fixation technique between outliers and surgeons below the outlier threshold for both revision rate and 6-month Oxford score using funnel plot control limits.

**Table 6:** Distribution of demographic factors and fixation for the 2-year timeframe. Surgeons were identified as outliers using the 95% upper control limit for revision rate and the 95% lower control limit for Oxford score. Values are percentages unless otherwise specified

	Revision rate at 2 years		6-month Oxford score	
	Outlier n = 969 ^a^	Within control n = 36,510 ^a^	Outlier n = 1,360 ^a^	Within control n = 36,119 ^a^
Sex				
Male	51	48	49	49
Female	49	52	51	51
Age (mean)	68.0	68.1	68.4	68.1
BMI (mean)	31.7	31.4	31.8	31.4
ASA class				
1	12	10	8.0	10
2	59	64	59	64
3	29	26	33	26
4	0.1	0.4	0.5	0.4
Ethnicity				
European	72	85	83	84
Māori	14	6.6	12	6.6
Pacifica	7.6	4.1	3.0	4.2
Asian	5.1	4.1	1.5	4.2
Diagnosis				
Osteoarthritis	98	96	97	96
Rheumatoid	1.2	1.7	2.1	1.7
Dysplasia	0.4	1.3	0.5	1.3
Fracture	0.9	1.1	1.4	1.1
Avascular necrosis	0.1	0.3	0.3	0.2
Tumor	0.0	0.1	0.0	0.1
Cementing				
Cemented	67	93	73	93
Uncemented	0.0	4.9	27	4.0
Hybrid	33	2.0	0.4	2.9

a Data for sex, age, ASA class, ethnicity, diagnosis, and cementing was available for > 99% of procedures. Body mass index (BMI) data was available for 28,883 procedures (77%) overall for this reporting timeframe.

**Table 7:** distribution of demographic factors and fixation for the 5-year timeframe. Surgeons were identified as outliers using the 95% upper control limit for revision rate and the 95% lower control limit for Oxford score. Values are percentages unless otherwise specified

	Revision rate within 5 years		6-month Oxford score	
	Outlier n = 4,270 ^a^	Within control n = 67,533 ^a^	Outlier n = 4,758 ^a^	Within control n = 67,045 ^a^
Sex				
Male	51	51	48	49
Female	49	49	52	51
Age (mean)	67.4	68.1	69.1	68.0
BMI (mean)	31.7	31.4	32.3	31.4
ASA class				
1	12	10	7.0	10
2	61	64	59	64
3	27	26	34	26
4	0.2	0.4	0.7	0.4
Ethnicity				
European	81	84	75	85
Māori	10	6.4	9.9	6.4
Pacifica	5.1	4.1	6.7	4.0
Asian	3.0	4.1	6.5	3.9
Diagnosis				
Osteoarthritis	97	96	97	96
Rheumatoid	1.6	1.9	2.0	1.8
Dysplasia	1.0	1.4	0.7	1.4
Fracture	0.9	1.1	0.8	1.2
Avascular necrosis	0.2	0.3	0.3	0.3
Tumor	0.0	0.1	0.1	0.1
Cementing				
Cemented	83	92	73	93
Uncemented	2.3	5.5	13	4.8
Hybrid	15	2.3	14	2.2

a Data for sex, age, ASA class, ethnicity, diagnosis, and cementing was available for > 98% of procedures. Body mass index (BMI) data was available for 54,591 procedures (76%) overall for this reporting timeframe.

**Table 8:** Distribution of demographic factors and fixation for the 10-year timeframe. Surgeons were identified as outliers using the 95% upper control limit for revision rate and the 95% lower control limit for Oxford score. Values are percentages unless otherwise specified

	Revision rate within 10 years		6-month Oxford score	
	Outlier n = 11,370 ^a^	Within control n = 95,787 ^a^	Outlier n = 9,693 ^a^	Within control n = 97,464 ^a^
Sex				
Male	51	48	47	49
Female	49	52	53	51
Age (mean)	67.5	68.2	69.1	68.0
BMI (mean)	31.7	31.4	32.1	31.4
ASA class				
1	11	10	8.8	11
2	63	64	57	64
3	26	26	33	25
4	0.4	0.4	0.7	0.4
Ethnicity				
European	82	85	79	85
Māori	8.8	5.6	6.6	5.9
Pacifica	5.2	3.7	6.7	3.6
Asian	2.8	3.7	5.5	3.4
Diagnosis				
Osteoarthritis	96	96	97	96
Rheumatoid	2.0	2.3	1.8	2.3
Dysplasia	1.1	1.1	0.5	1.2
Fracture	1.1	1.1	0.7	1.2
Avascular necrosis	0.3	0.3	0.3	0.3
Tumor	0.0	0.1	0.0	0.1
Cementing				
Cemented	89	92	70	94
Uncemented	2.3	4.7	17	3.2
Hybrid	9.0	3.4	13	3.1

a Data for sex, age, ASA class, ethnicity, diagnosis, and cementing was available for > 98% of procedures. Body mass index (BMI) data was available for 54,591 procedures (76%) overall for this reporting timeframe.

Outliers for revision at 2 years had a higher proportion of hybrid fixation, Māori ethnicity, and Pacifica ethnicity compared with surgeons below the outlier threshold (P < 0.001). Outliers for 6-month Oxford score for this timeframe had a higher proportion of uncemented fixation, Māori ethnicity, and ASA class 3 or 4 compared with surgeons below the outlier threshold (P < 0.001).

Outliers for revision within 5 years had a higher proportion of hybrid fixation, Māori ethnicity, and Pacifica ethnicity compared with surgeons below the outlier threshold (P < 0.001). Outliers for 6-month Oxford score for this timeframe had a higher proportion of uncemented fixation, Māori ethnicity, Pacifica ethnicity, Asian ethnicity, and ASA class 3 or 4 compared with surgeons below the outlier threshold (P < 0.001).

Outliers for revision within 10 years had a higher proportion of hybrid fixation, Māori ethnicity, and Pacifica ethnicity compared with surgeons below the outlier threshold (P < 0.001). Outliers for 6-month Oxford score for this timeframe had a higher proportion of uncemented fixation, hybrid fixation, Māori ethnicity, Pacifica ethnicity, Asian ethnicity, and ASA class 3 or 4 compared with surgeons below the outlier threshold (P = 0.005 for Māori ethnicity, otherwise P < 0.001).

Across all timeframes, there was a tendency for revision rate outlier surgeons to be associated with hybrid fixation, and Māori and Pacifica ethnicity. Outlier surgeons for 6-month Oxford score tended to be associated with uncemented fixation, higher ASA class, and Asian ethnicity, in addition to these factors. Age, sex, BMI, and preoperative diagnosis were equally distributed between outliers and non-outliers for revision rate and Oxford score for all 3 timeframes.

## Discussion

This is the first study to compare potential outlier performance following total knee arthroplasty using revision rates and patient-reported outcome measures.

There was minimal overlap between potential outliers identified using centile thresholds or funnel plot control limits. Better correlation between outliers identified using the 2 methods had been anticipated and poor correlation raises questions regarding the most appropriate method for measuring and reporting surgeon-level arthroplasty outcomes. Revision rates are commonly used for this purpose; however, not all patients with a poor outcome following total knee arthroplasty require a revision procedure [7]. The findings of this study, that surgeons with low mean 6-month Oxford scores do not tend to have meaningfully higher revision rates, suggests reporting revision rates alone may result in poor outcomes being underrepresented for some surgeons. Patient-reported outcome measures provide a broader understanding of surgeons’ outcomes [8]. However, reporting patient-reported outcome measures alone risks results being perceived as subjective by surgeons and a combined approach may be preferable. A recent survey of arthroplasty surgeons in New Zealand supported the use of patient-reported outcome measures alongside revision rates [5].

The findings of the additional analysis on 5-year Oxford scores provides reassurance that minimal overlap in outliers is not limited to the 6-month score. 5 years is the second timepoint for Oxford score collected in the NZJR and comparison with revision within 10 years was selected as it remained consistent with the theme of evaluating Oxford score as a predictor of revision. We preferred the 6-month Oxford score for evaluating surgeon performance, as this facilitates timely feedback to surgeons and ensures results reflect recent practice.

The finding relating to the association between revision rate and Oxford score at the patient level is consistent with the published literature. Rothwell et al. previously reported the association between Oxford score at 6 months and risk of revision at 2 years using the New Zealand Joint Registry [9]. The figure reported in this study is consistent with that reported by Rothwell et al. of a 9.9% increase in risk of revision for each 1 point decrease in Oxford score (P < 0.001). The association remained statistically significant at the surgeon level for the included reporting timeframes; however, the strength of association was weak. A stronger association between 6-month Oxford score and risk of revision at the surgeon level had been anticipated. Further research, comparing Oxford scores with revision rates in other populations, would be helpful to confirm this finding.

Critics of patient-reported outcome measures point to their subjective nature and concern regarding completeness of data, low response rates, and reliability [10-12]. Concerns have also been raised that patient-reported outcome measures may reflect a patient’s level of satisfaction with their care as opposed to outcomes. However, Black et al. demonstrated poor correlation between patient satisfaction and patient-reported outcome measures, suggesting this concern is misguided [13]. Hamilton and colleagues have argued that “distrust” of patient-reported measures is also misguided as many are associated with high reliability and more objective than critics suggest [11]. Hahn et al. previously compared the reliability of patient-reported outcome measures with common clinical measurements and demonstrated favorable patient-reported outcome measure performance [14]. The New Zealand Joint Registry has a good response rate and relatively complete data for Oxford Score at 6 months.

There is lack of consensus on the most appropriate patient-reported outcome measure for monitoring performance in total knee arthroplasty. The Oxford hip and knee scores are widely used, well regarded, and have been demonstrated to be amongst the best performing arthroplasty-specific patient-reported outcome measures [15,16]. One potential problem is the significant ceiling effect postoperatively, where a large proportion of patients achieve the maximum possible score [17]. NZJR data demonstrates that 39% of respondents had an Oxford knee score greater than or equal to 42 out of 48 at 6 months after primary knee arthroplasty, corresponding to an excellent outcome [7]. However, given that the purpose of surgeon-specific outcome monitoring is to identify suboptimal performance, this is less problematic and would be preferred to a measure with a significant floor effect. In our opinion, the Oxford score is a good measure for comparing surgeon-level performance in the context of quality assurance. The findings of this study are specific to the Oxford knee score and it is uncertain whether the lack of correlation would translate to other patient-reported outcome measures or procedures.

The relationship between total knee arthroplasty fixation technique and outlier surgeons for both revision rate and Oxford score demonstrated in this study is interesting. A difference between fixation techniques has not been consistently demonstrated in the literature. However, this study suggests hybrid and uncemented fixation are more frequently used by outlier surgeons for Oxford score, and hybrid fixation is more frequently used by outliers for revision rate. This could have implications when developing strategies to help outlier surgeons improve their practice; these surgeons could consider changing to cemented fixation or, at least, establish what is causing hybrid or uncemented fixation to fail at a higher rate in their practice. Māori and Pacific populations represent minority ethnic groups in New Zealand with generally poorer health outcomes than the majority European population. The finding that outlier surgeons tend to operate on a higher proportion of patients from these ethnic groups is consistent with this. In addition, it is not unexpected that outlier surgeons for 6-month Oxford score tend to operate on a higher proportion of ASA class 3 and 4 patients. These patients have a greater number, and severity, of comorbidities, which have the potential to limit rehabilitation. It is notable that this did not translate to revision rate outliers. The association between demographic factors and being identified as an outlier emphasizes the importance of considering case-mix when evaluating surgeon performance.

### Strengths

We included high-quality data. Prospectively collected data on outcomes was included for a large number of surgeons and patients. The quality and completeness of data held by the New Zealand Joint Registry is good, affording a high degree of confidence in the findings of this study [5]. One study published by Zhu et al. in 2016 reported incomplete capture of revisions secondary to infection in 1 region, although their definition included all reoperations for infection whereas the registry definition requires a component be exchanged [18]. No other concerns have been raised regarding quality of data collected by the New Zealand Joint Registry. It is important to recognize that, due to the high number of joints in the registry, questionnaires are sent to a randomly selected group of patients, targeting an annual response rate of 20%. This accounts for differences in the scales for the Oxford score and revision rate funnel plots included in Figures 4 to 6.

### Limitations

The inability to consider change in Oxford scores, due to NZJR not collecting preoperative scores, is a limitation of this study. It is known that preoperative Oxford score predicts postoperative score and it is possible that some surgeons identified as outliers have acceptable change in Oxford scores [19]. This represents an area for further research. Another limitation of the analysis in this study is absence of alternative shorter-term Oxford score timeframes, such as 1 or 2 years, in the NZJR. These represent reasonable alternatives to the 6-month timeframe and could have furthered the analysis in this study. However, the 6-month Oxford score is feasible for evaluating performance and allows more expeditious feedback to surgeons.

### Conclusion

We showed that the 6-month Oxford score identifies different outliers to revision rate. Lack of correlation between potential outliers identified using revision rate and 6-month Oxford score highlights the importance of taking a broad approach to monitoring surgical performance.

*In perspective,* the findings of this study support the use of 6-month Oxford score, alongside revision rate, to monitor surgeon-level outcomes.

## Notes

ML: conceptualization, study design, analysis, interpretation, draft and final manuscript. CF: study design, analysis, interpretation, final manuscript, supervision. GH: conceptualization, study design, interpretation, final manuscript, supervision.

Handling co-editor: Per Henrik Randsborg

*Acta* thanks Alexander David Liddle and Bart G Pijls for help with peer review of this manuscript.
